# Surface functionality analysis by Boehm titration of graphene nanoplatelets functionalized *via* a solvent-free cycloaddition reaction†

**DOI:** 10.1039/c8na00280k

**Published:** 2019-01-18

**Authors:** He Ren, Eunice Cunha, Quanji Sun, Zheling Li, Ian A. Kinloch, Robert J. Young, Zhaodong Fan

**Affiliations:** Beijing Institute of Aeronautical Materials (BIAM) Beijing China fanzhaodong99@sohu.com; National Graphene Institute, School of Materials, University of Manchester Manchester M13 9PL UK robert.young@manchester.ac.uk

## Abstract

In this work, the functionalization of graphene nanoplatelets (GNPs) performed by a solvent-free cycloaddition reaction on GNPs with iminodiacetic acid (IDA) and paraformaldehyde (PFA), and the functionality analysis of the resulting functionalized GNPs (f-GNPs) by Boehm titration are introduced. The f-GNPs synthesized at different temperatures were characterized by X-ray diffraction (XRD), Raman spectroscopy and scanning electron microscopy (SEM) for structural and morphological properties. Back titration of the f-GNPs selectively identified 3 types of functional groups on the f-GNP surface, carboxylic, lactonic and phenolic, and suggested that 200 °C gives the highest carboxylic group functionality. With the reaction temperature increasing from 180 to 220 °C, a decrease in the phenolic functionality and an increase in that of lactonic are observed. In the case of 250 °C reactions, it was found that the carboxylic functionality is greatly reduced, while the phenolic functionality showed a significant increase. The f-GNP samples were further characterized by thermogravimetric analysis (TGA) and X-ray photoelectron spectroscopy (XPS), the results of which showed a good agreement with the titration analysis.

## Abstract 2

Carboxylic acid-terminated pyrrolidine functionalities were covalently bonded to the surface of graphene nanoplatelets *via* a solvent-free approach and characterized by Boehm titration.

## Graphical abstract

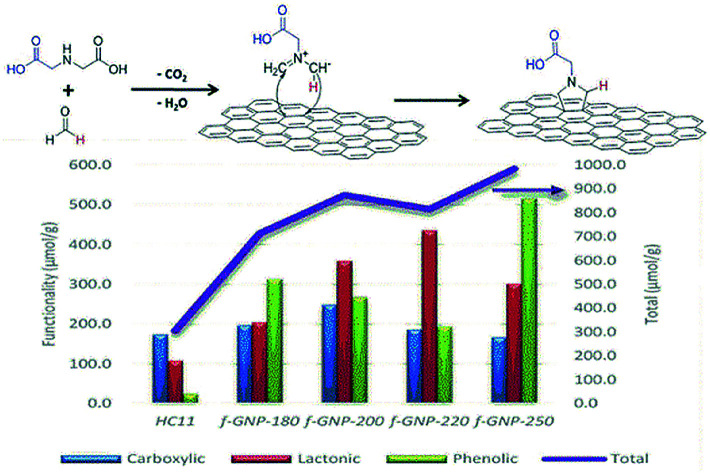

## Introduction

The increasing research over the past decade into the excellent properties that graphene possesses has led to fruitful outputs that have demonstrated a wide range of potential applications, where graphene and its composites have played a remarkable role.^1–3^ Surface modification of graphene is a practical method that could on the one hand alter the surface properties, while on the other hand, introduce functional groups covalently/non-covalently into graphene sheets, so that the desired performance could be realized.^4–6^ It is by such modification that the applications of graphene-based materials in the fields of solar cells, supercapacitors, biomedical applications, gas storage, composites and others have been developed.^7–11^

Among all the covalent functionalization methods, the 1,3-dipolar cycloaddition (DCA) of azomethine ylides, which was first introduced by Huisgen in 1963, is one of the reactions that can take place under mild conditions and provide heterocyclic products with little breakage of carbon nanotubes (CNTs) at a high yield.^12–14^ It has been applied in the functionalization of various carbon-based materials, such as fullerenes (C_60_s),^15,16^ CNTs,^17–19^ and graphene.^4,20,21^ In 2010, when Trapalis *et al.* published their work on applying the 1,3-DCA reaction to exfoliated graphene, this reaction was introduced for the first time in the studies of covalent functionalization of graphene.^20^ The mechanism and the reactive sites on graphene sheets where the reaction will typically take place, as well as the functional group density of functionalized graphene, have been studied intensively. It is generally accepted that reactions take place mainly at the edges of graphene sheets and defects located on basal planes, although some details are still not clear or are presented with contradictory conclusions.^22–24^

Traditionally, 1,3-DCA reactions are performed in the liquid phase. Since graphene is not soluble in typical solvents used for DCA, a low graphene concentration and an extended reaction time can be expected for a better distribution within the suspension and higher degree of reaction. However, such a low graphene concentration may also lead to a limited reaction scale and efficiency.^5^ A solvent-free approach is considered as a possible solution to realize the scaling up of graphene functionalization *via* DCA. A solid state 1,3-DCA reaction has been performed in the functionalization of CNTs by applying microwave irradiation.^25^ It is noteworthy that the application of microwaves as an energy source is complex and it is difficult to control the temperature and other reaction conditions. The approach reported by Paiva *et al.* could be an alternative, in which the solvent-free functionalization of CNTs was performed with *N*-benzyloxycarbonyl glycine and formaldehyde.^26^

Graphite nanoplatelets or graphene nanoplatelets (GNPs) are derived from graphite flakes.^27^ They are typically platelets containing a few (typically, tens) layers of sp^2^ carbon packed in the manner of graphite, with thicknesses up to approximately 100 nm. Prepared by heat or radiation-induced expansion of graphite, GNPs are more economically viable for large-scale production, compared to graphene, due to the relaxed requirements for the number of layers. Admittedly, the increase in the interlayer spacing of GNPs compared to graphite leads to a weakened van der Waals interaction between the sheets within GNPs, and furthermore, the multi-layered nature of GNPs makes them uncompetitive with single layered graphene in terms of physical properties, but the compromise between performance and economic viability makes GNPs a promising candidate for real applications.^5,27–29^

Possible applications of GNPs and the processing techniques required can be considered as analogous to those of graphene, where appropriate functionalization is often desired. Our group has recently studied the solvent-free 1,3-DCA reaction of GNPs with IDA and PFA, by which time-efficient and scalable functionalization of GNPs can be realized.^30^

The determination of functionality is important for the surface modification of GNPs, especially where further quantitative reactions or functional group density sensitivity studies are to be performed. Considering the insoluble nature of GNPs and their derivatives in common solvents, Boehm titration can be applied as an effective method for characterizing the oxygen-containing functional groups of f-GNPs.^31–33^ In principle, Boehm titration works on the basis that different types of oxygen-containing groups have different acidities and can be selectively neutralized with different bases, namely sodium bicarbonate (NaHCO_3_, p*K*_a_ = 6.37), sodium carbonate (Na_2_CO_3_, p*K*_a_ = 10.25) and sodium hydroxide (NaOH, p*K*_a_ = 15.74). Carboxylic groups (p*K*_a_ = 3–6), lactonic groups (p*K*_a_ = 7–9) and phenolic groups (p*K*_a_ = 8–11) are the three major types of oxygen-containing groups on carbon materials.^31,34^ It is suggested that NaOH can neutralize all three types, Na_2_CO_3_ the first two, and sodium bicarbonate only carboxylic groups. Therefore, the quantification of acidic groups can be readily realized by monitoring the equivalence reactions between these groups and the applicable base.

In this work, the functionalization of GNPs through the 1,3-DCA reaction in the solid state was performed at selected temperatures of 180 °C, 200 °C, 220 °C and 250 °C. By introducing covalently bonded carboxylic acid-terminated pyrrolidine groups mainly to the edges and surface defects of GNPs, ‘virtually defect-free’ f-GNPs have been synthesized.^20^ These f-GNPs with less structural defects could be an alternative to graphene oxide in applications such as composites, biomedical systems and biosensors, where carboxylic groups are made use of as reactive sites.^20,35–38^ Boehm titration was employed to study the functionality of carefully washed products. By comparing the functionality of f-GNP samples, it is revealed that the functionality of f-GNPs synthesized *via* the solid-state DCA reaction introduced herein is strongly temperature dependent. These results can be correlated with and are in good agreement with TGA and XPS results. Studies of their morphological and structural properties were also conducted by employing different characterization techniques.

## Experimental

### Materials

GNPs (grade Micrograf HC11) were provided by Nacional de Grafite, Brazil; IDA (purity ≥ 98%), PFA, NaHCO_3_, NaOH, and potassium hydrogen phthalate (KHP), of reagent grade, were purchased from Sigma Aldrich; absolute ethanol (EtOH) was purchased from VWR Chemicals; acetone and Na_2_CO_3_, of analytical reagent grade, were purchased from Fisher Scientific; hydrochloric acid (HCl, fuming, 37%) was purchased from Honeywell Fluka. All chemicals/reagents were used as received.

### Functionalization of the GNPs

The functionalization of the GNPs was performed following the cycloaddition reaction scheme demonstrated by a procedure previously reported.^26^ Iminodiacetic acid (IDA) and paraformaldehyde (PFA) (IDA : PFA, molar ratio 1 : 5) were dissolved in ethanol under magnetic stirring in a 3-neck round bottom flask, followed by the addition of the GNPs HC11 (HC11 : IDA, measured in weight, 1 : 1). The suspension was heated gently until the ethanol was completely removed. The resulting solid mixture was broken down to a fine powder and heated at selected temperatures (180 °C, 200 °C, 220 °C and 250 °C) under reflux for 5 h, in the same flask. The product was collected in a capped bottle and re-dispersed in a mixed solvent of water, acetone and ethanol by sonication for 20 min. The suspension was filtered and washed sequentially with water (until the filtrate became colourless), acetone (3 times) and ethanol (3 times). The filter residue, f-GNPs, was dried under vacuum overnight at 40 °C.

### Characterization

#### X-ray diffraction and scanning electron microscopy

Samples (HC11 and f-GNPs) in the powder form were analysed by XRD with a PANalytical X'Pert Pro system equipped with a PW3050/60 goniometer. The X-ray source was a sealed copper tube operated at 40 keV and 40 mA.

Samples were studied for morphological properties using a SEM-Zeiss Sigma VP FEG-SEM microscope operating at 8 kV.

#### Raman spectroscopy

Raman characterization was performed on a Horiba Labram HR Evolution confocal microscope, equipped with a motorized *X*–*Y* stage, using an excitation laser of 633 nm and a 100× objective lens. Samples were prepared by drop casting the HC11 and f-GNP ethanol suspension on a glass slide and subsequent drying on a hot plate.

#### Thermogravimetric analysis and X-ray photoelectron spectroscopy

TGA was performed for each sample using a TA Instruments® Modulated TGA Q5000, with a heating rate of 10 °C min^−1^ (1) under a constant flow of N_2_ and (2) in air.

XPS was performed on a Kratos Axis Ultra X-ray photoelectron spectrometer; data treatment and curve fitting were accomplished using CasaXPS and OriginPro® software.

#### Electrical conductivity

For the electrical conductivity measurement, the HC11 and f-GNPs powders were pressed, at 10 tons for 5 minutes, into pellets (13 mm in diameter and around 0.8 mm in thickness). The samples were analysed using a Jandel RM3000 four-point probe system.

#### Zeta potential

Zeta potential analysis was performed using a Zetasizer Nano ZS instrument (Malvern Instruments, UK). At least three zeta potential measurements were performed for each sample. The results are presented as the average of these measurements.

#### Boehm titration general procedure

The functionality of the pristine HC11 material and the f-GNPs synthesized at different temperatures was studied by Boehm titration in an indirect manner, denoted in the literature as back titration.^31^ 0.75 g of GNPs (pristine HC11 or f-GNPs) was added to 50 mL 0.05 M aqueous solution of one of the three selected bases: NaHCO_3_, Na_2_CO_3_ or NaOH. For each material and selected base, 3 parallel samples were prepared. The samples were agitated by magnetic stirring for 24 h and then filtered. Three aliquots with a volume of 10 mL were taken from the filtrate of each sample and acidified using HCl (0.05 M) with a volume of 15 mL (NaHCO_3_ and NaOH) or 30 mL (Na_2_CO_3_) for complete neutralization. The acidified solution was purged with N_2_ under magnetic stirring for 2 hours for CO_2_ expulsion. The aliquots were then titrated with 0.05 M NaOH (aq.) which was standardized with KHP solution (0.05 M/20 mL).

The potentiometric examinations were performed using a Hanna Instruments HI-991301 pH/EC/TDS/temperature meter, with a pH measurement accuracy of ±0.01. The meter was calibrated by a one-point calibration method with a standard pH 4.01 buffer. The end point, which determines the titration volume, is determined by reading data point “pH = 7” from the full potentiometric titration curve.

#### Surface functionality calculations

Since an indirect method (back titration) is adopted in this work, the carbon surface functionality (CSF) can be calculated using^31^1
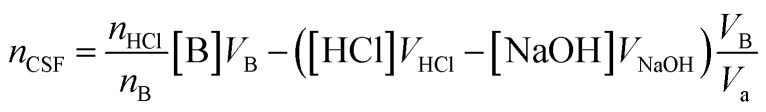
2
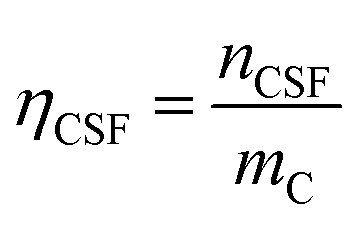
where *V*_HCl_, *V*_NaOH_, and *V*_B_, and [HCl], [NaOH] and [B], are the volume and concentration of HCl, NaOH and the reaction base added to each sample prepared, respectively, while *V*_a_ is the volume of the aliquot taken from *V*_B_. The CSF calculated is denoted as *n*_CSF_, in moles. *η*_CSF_, in mol g^−1^, represents the CSF per unit mass, which is *n*_CSF_ divided by the weight of carbon used in the preparation of each sample. It is noteworthy that, for NaHCO_3_ and NaOH, the neutralization reaction takes place with an acid/base ratio of 1 : 1, while that of Na_2_CO_3_ is 2 : 1. Therefore, in the calculation of reacting functional groups on the surface of HC11 and f-GNPs, the molar ratio of acid to base *n*_HCl_/*n*_B_ should be taken into consideration.^31^

The selective neutralization of different functional groups by different titrating bases enables the estimation of functionalities of these groups by simply calculating the difference of the *n*_CSF_ calculated for different titrating bases: the differences between *n*_CSF_ (NaHCO_3_) and *n*_CSF_ (Na_2_CO_3_), and between *n*_CSF_ (Na_2_CO_3_) and *n*_CSF_ (NaOH) give the functionalities of lactonic groups and phenolic groups, respectively.

## Results and discussion

**Scheme 1:**
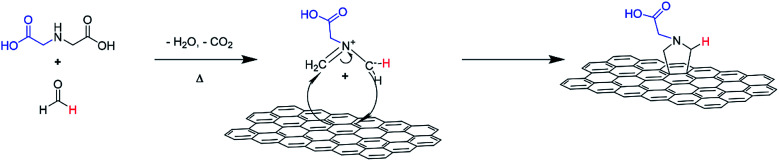
Schematic illustration of GNP functionalization *via* a 1,3-dipolar cycloaddition reaction.

An illustration of the cycloaddition reaction of HC11 with IDA and PFA is shown in Scheme 1. The functionalization of HC11 was performed *via* a solvent-free 1,3-DCA reaction. PFA degrades with heat and in the presence of H_2_O, generating formaldehyde which then facilitates the formation of 1,3-dipolar species (azomethine ylide). The 1,3-dipole reacted with HC11 following the cycloaddition scheme and facilitated the formation of a carboxylic acid-terminated pyrrolidine ring covalently bonded to the surface of the HC11, denoted as f-GNPs. The temperature dependence of the DCA reaction was studied by performing the reaction at different temperatures: 180, 200, 220 and 250 °C. The products, in the powder form, are denoted as f-GNPs-180, -200, -220 and -250, respectively. It was observed that due to reflux of evaporated reactants, the temperature of the mixture is varied within a range of approximately ±5 °C.

### Structural and morphological analysis

XRD, SEM and Raman microscopy were adopted to characterize the structural and morphological properties of pristine HC11 and the f-GNP products.

**Fig. 1:**
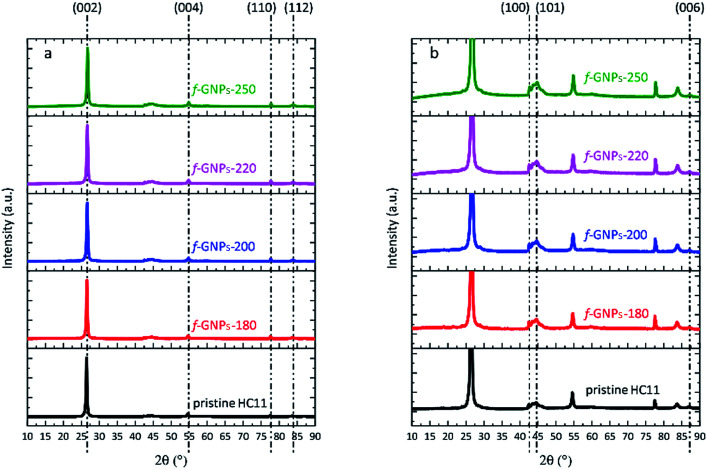
(a) XRD spectra of pristine HC11 and f-GNPs prepared at different temperatures; (b) rescaled spectra with regard to the *y*-axis of (a).

The XRD spectra of pristine HC11 and f-GNPs prepared at different temperatures are shown in Fig. 1(a). The diffraction patterns showed similarity to that of graphitic carbon. Diffraction peaks observed at 2*θ* = 26.5°, 54.6°, 77.5° and 83.7° are assigned to (002), (004), (110) and (112) planes, respectively. By rescaling the spectra, peaks are identified within a 2*θ* range from 40 to 50°, which further suggests a graphitic structure (Fig. 1(b)).^39^ In general, the spectrum of each sample is almost identical except that the f-GNPs show a more graphitic appearance.

**Fig. 2:**
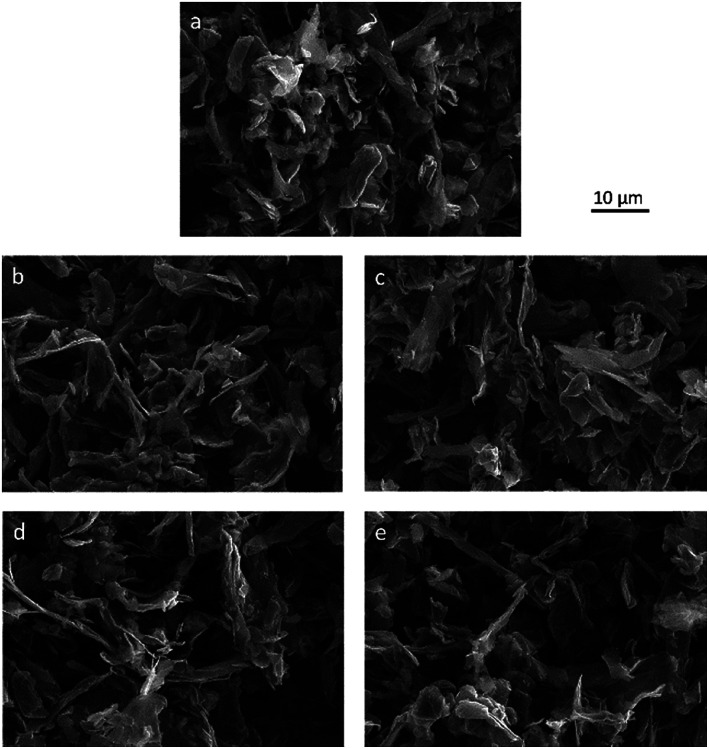
SEM images of (a) pristine HC11, (b) f-GNPs-180, (c) f-GNPs-200, (d) f-GNPs-220 and (e) f-GNPs-250.

The surface morphology of pristine and functionalized materials was studied by SEM. The SEM images are presented in Fig. 2(a)–(e). The morphology of GNP flakes with lateral dimensions of approximately 10 μm can be identified. Compared to pristine HC11 (Fig. 2(a)), the change in the morphology of the functionalized GNPs (Fig. 2(b)–(e)) is marginal.

**Fig. 3:**
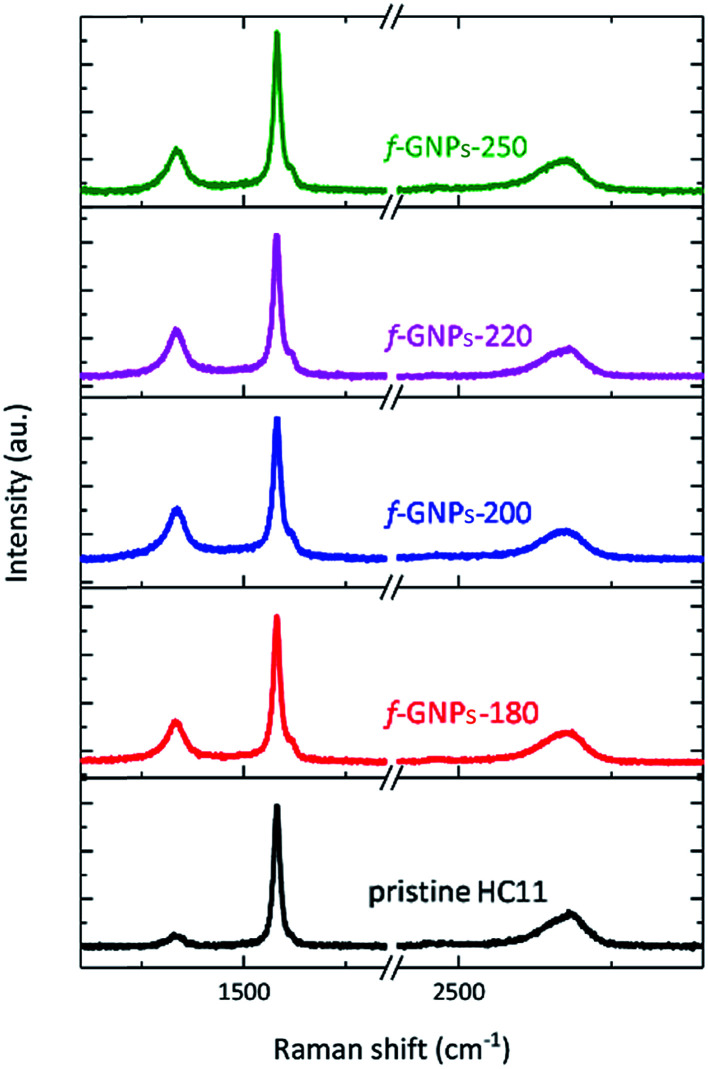
Raman spectra of pristine HC11 and f-GNPs.

Fig. 3 presents the Raman spectra of the pristine HC11 and f-GNPs. In each spectrum, three main characteristic bands typical for graphitic materials are identified: the D band (∼1330 cm^−1^), the G band (∼1580 cm^−1^) and the 2D band (∼2680 cm^−1^).^40,41^ Analysis of the peak position, intensity and shape provides information on the sp^2^ hybridized carbon, strain, crystallinity and number of stacked carbon layers, (the G and 2D band), and sp^3^ carbon within defect regions or covalently bonded functional groups (the D band).^40–42^ As is shown in Fig. 3, the f-GNPs clearly show a more prominent D band and a shoulder in the G band at around 1620 cm^−1^, related to the D′ band, indicating the formation of sp^3^ bonds.^43^ Furthermore, an increase of the area of the D band normalized by the area of the G band (*A*_D_/*A*_G_) is observed along with the increase of the full width at half-maximum (FWHM) of the G band for the f-GNPs (Table 1). This indicates the formation of an arbitrary amount of sp^3^ hybridized carbon resulting from the functionalization.^40,44–46^ The area of the 2D band normalized by the area of the G band (*A*_2D_/*A*_G_) is not much different from that of pristine HC11 suggesting that the structural quality of the f-GNPs is not greatly affected by the functionalization.

**Table 1:** *A*_D_/*A*_G_, FWHM and *A*_2D_/*A*_G_ calculated from the Raman spectra of the GNPs

	*A*_D_/*A*_G_	FWHM (cm^−1^)	*A*_2D_/*A*_G_
GNPs	0.4 ± 0.1	19 ± 1	0.36 ± 0.13
f-GNPs-180	0.6 ± 0.1	20 ± 1	0.26 ± 0.12
f-GNPs-200	0.7 ± 0.2	21 ± 2	0.33 ± 0.05
f-GNPs-220	0.7 ± 0.1	22 ± 2	0.27 ± 0.07
f-GNPs-250	0.6 ± 0.1	21 ± 2	0.25 ± 0.06

The electrical conductivity of pristine HC11 and f-GNPs was also measured using a four-point probe system. The electrical conductivity was found to be 298 ± 3 S m^−1^, 269 ± 2 S m^−1^, 259 ± 3 S m^−1^, 253 ± 2 S m^−1^ and 252 ± 2 S m^−1^ for HC11, f-GNPs-180, f-GNPs-200, f-GNPs-220 and f-GNPs-250, respectively. These results show that there is a small decrease in the electrical conductivity going from HC11 to the f-GNPs, although all the values are of the same order of magnitude. This indicates that the functionalization did not significantly affect the structural properties of the pristine HC11, in agreement with the Raman and XRD analysis.

The stability of the aqueous f-GNP suspensions was characterized by zeta potential (*ζ*) measurements. f-GNPs-180, f-GNPs-200, f-GNPs-220 and f-GNPs-250 have *ζ* values of −42 ± 1 mV, −41 ± 2 mV, −42 ± 2 mV and −43 ± 2 mV, respectively, which indicates that the f-GNPs are all capable of forming stable suspensions in water owing to the strong electrostatic repulsion between them.^47,48^

### Surface functionality determination by Boehm titration

**Fig. 4:**
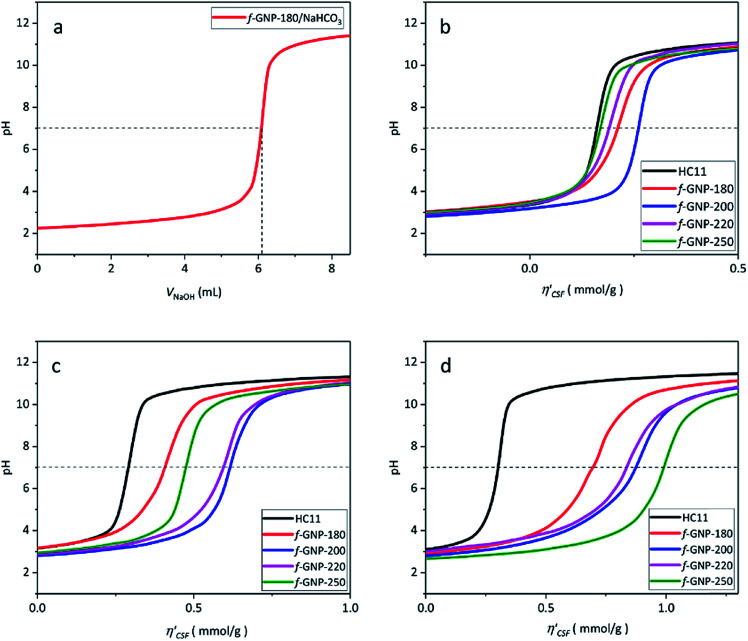
(a) Titration curve of f-GNPs-180/NaHCO_3_ with NaOH; (b)–(d) 
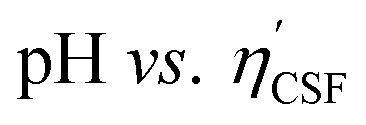
 calculated from the *V*_NaOH_ of pristine HC11 and functionalized GNPs where NaHCO_3_, Na_2_CO_3_ and NaOH were applied as selective reacting bases, respectively.

**Fig. 5:**
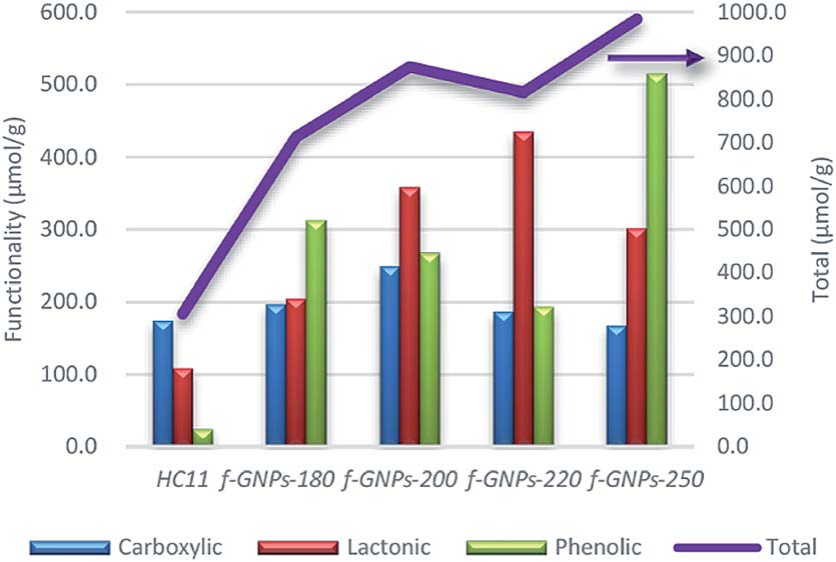
The comparison of the functionality (in μmol g^−1^) of different oxygen-containing functional groups and the *η*_CSF_ (NaOH) of pristine HC11 and that of f-GNPs produced at different temperatures.

Fig. 4(a) shows an illustration of a typical titration curve obtained, where NaHCO_3_ was used as the selective reacting base. Given that *V*_NaOH_ can be correlated with CSF using eqn (2), the titration process can be presented by plotting 
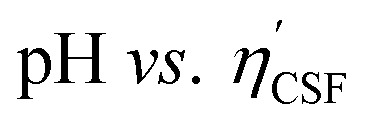
 instead of pH *vs. V*_NaOH_ to favour the comparison between different carbon samples. The 
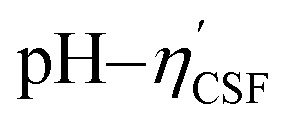
 curves for different reacting bases are shown in Fig. 4(b)–(d). The *η*_CSF_ of each GNP sample can be found at the intercept of the dashed line (pH = 7.0) with each curve and is summarized in Table 2. The functionalities of the three major classes (carboxylic, lactonic and phenolic) can thus be calculated and are compared in Fig. 5. As shown in Fig. 5, the carboxylic group content of the f-GNPs undergoes an increase and then decreases with the reaction temperature increasing from 180 °C to 250 °C. A maximum functionality can be expected for f-GNPs-200. The lactonic and phenolic group contents compensate each other at reaction temperatures of 180, 200 and 220 °C: the increase in the reaction temperature leads to an increase in the lactonic functionality and a decrease in phenolic groups; however this pattern was reversed when the reaction temperature increased from 220 to 250 °C.

**Table 2:** *η*_CSF_ calculated from titration experiments with different reacting bases of pristine HC11, f-GNPs-180, f-GNPs-200, f-GNPs-220 and f-GNPs-250 (in μmol g^−1^)

	HC11	f-GNPs-180	f-GNPs-200	f-GNPs-220	f-GNPs-250
*η*_CSF_ (NaHCO_3_)	166 ± 20	197 ± 23	248 ± 18	186 ± 11	167 ± 12
*η*_CSF_ (Na_2_CO_3_)	282 ± 32	401 ± 8	606 ± 13	621 ± 32	468 ± 23
*η*_CSF_ (NaOH)	306 ± 2	714 ± 23	874 ± 5	815 ± 9	983 ± 23

Vinciguerra *et al.* have demonstrated the thermal behaviour of IDA in their previous work.^49^ The work suggested that IDA undergoes a mass loss, which is attributed to the loss of water and the formation of iminodiacetic anhydride, at 217–252 °C. During this process, little evolution of CO_2_ can also be observed, which is considered as an indication of a partial decarboxylation process. An even more complex thermal degradation mechanism was reported by González Vílchez *et al.*, where the increasing temperature promotes the breaking of hydrogen bonds and the loss of 2H_2_O molecules per 2 bonded IDA molecules.^50^ Furthermore, in the work of the functionalization of carbon nanotubes *via* DCA demonstrated by Paiva, *et al.*, thermal degradation of the amine at a temperature of around 210 °C may also lead to a change in the resulting functional groups.^26^ The study regarding esterification of carboxylic acids in solid state reactions by Pantze, *et al.* revealed the esterification reaction rate dependence on the reaction temperature, acid reactivity, pH, *etc.* It is found that in the solid state, the esterification may take place at temperatures above 150 °C and that the rate would increase and then decrease after reaching a maximum. In the herein reported DCA reaction, with increasing the reaction temperature from 180 to 220 °C, the functionalities of lactonic and phenolic groups change monotonically as a consequence of the increasing esterification rate, while those of carboxylic groups increase and then decrease because of the balance between the yield of DCA and the consumption by esterification. When the reaction temperature increased beyond a maximum, probably ∼220 °C, the esterification reaction rate dropped, and the carboxylic group yield also decreased due to unfavourable degradation. This leads to a drop in the functionality of both carboxylic and lactonic groups and a dramatic increase in that of phenolic groups, which is assumed to be the second reactant of the esterification reaction other than carboxylic groups.^51^

The thermal degradation of IDA may be a good explanation for the change in functionalities with the change of reaction temperature. The increase in temperature may favour the cycloaddition reaction; however, when the temperature goes beyond 210 °C, unexpected thermal degradation will lead to decarboxylation of the carboxylic group as well as dehydration and further degradation of IDA. This will lead to a reaction mechanism that may occur simultaneously with the 1,3-DCA reaction shown in Scheme 1.

### Thermal gravimetric analysis and X-ray photoelectron spectroscopy studies

Although titration results only provided general information on the molar content of three typical types of oxygen-containing groups, and therefore not necessarily representing the weight percentage of the corresponding functional groups, the TGA results may still provide evidence to estimate and support functional group contents calculated for them.

**Fig. 6:**
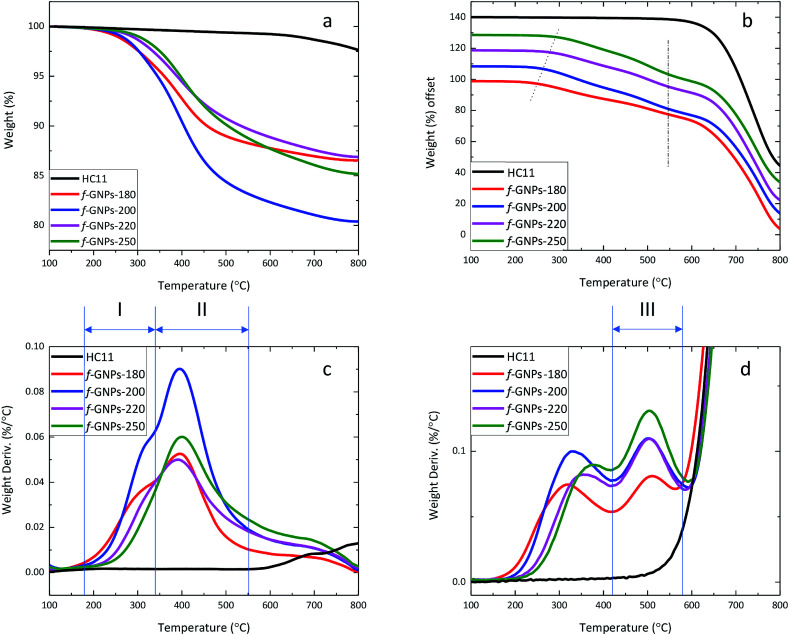
Thermal behaviour of the GNPs: (a) TGA curves of GNPs under an N_2_ stream; (b) TGA curves of the GNPs in air; (c) weight derivation against temperature of GNPs under an N_2_ stream; (d) weight derivation against temperature of the GNPs in air.

By analysing the thermal degradation behaviour (Fig. 6) of the GNPs, information on the weight percentage of functional groups with different thermal stabilities can be obtained. The weight loss of pristine HC11 and f-GNPs at 300 °C and 600 °C (1) under a N_2_ flow and (2) in air is summarized in Table 3, where f-GNPs-200 show the highest total weight loss, followed by f-GNPs-250, -180 and -220. The TGA curves and the weight derivation against temperature curves of pristine HC11 and f-GNPs tested under a N_2_ flow are shown in Fig. 6(a) and (c), respectively. The curves (f-GNPs-180, -200 and -220) shown in Fig. 6(c) can be resolved to two major peaks: stage (I) 180–345 °C and stage (II) 345–550 °C. Degradation stage (I) is attributed to the decarboxylation of the carboxylic groups (and some functional groups with lower stability for f-GNPs-180). Therefore, the weight loss can be correlated to the carboxylic functionalities of the f-GNPs, which is in good agreement with the titration results. Degradation stage (II) could result from the cleavage of the methyl group in the *N*-methyl-pyrrolidine functionalized GNPs after the decarboxylation of carboxylic groups.^52^

**Table 3:** Summary of TGA weight loss at 300 and 600 °C in N_2_ and air, and at 430 °C in air of the GNP samples

	300 °C in N_2_ (%)	300 °C in air (%)	600 °C in N_2_ (%)	600 °C in air (%)	430 °C in air (%)
HC11	0.28	3.33	0.76	3.33	0.57
f-GNPs-180	2.41	5.82	11.61	25.47	14.16
f-GNPs-200	2.36	5.92	16.2	31.17	17.13
f-GNPs-220	1.33	3.61	11.02	27.44	13.49
f-GNPs-250	0.98	3.09	12.12	29.89	13.45

The thermal degradation behaviour of pristine HC11 and f-GNPs was also studied in air (Fig. 6(b) and (d)). Similar to those in N_2_, the decarboxylation and the cleavage of methyl groups can be observed. The degradation onset shifted to the right (as is indicated by the dotted line in Fig. 6(b)) with the increase of reaction temperature. The difference in relatively less thermally stable groups' content can be considered as the major cause of this shift. A third stage of degradation can be found for all four f-GNPs at 430–580 °C in Fig. 6(d). This may indicate further degradation of the pyrrolidine groups. The inflection point of each curve at ∼575 °C, emphasized by the dash-dot line (Fig. 6(b)), suggests that the completion of the pyrrolidine group decomposition dominated the weight loss process. Zeroing the weight loss at 430 °C, the calibrated weight loss at 600 °C of f-GNPs-180, -200, -220, and -250 is 13.18%, 16.94%, 16.13% and 18.99%, respectively.

Thermal analysis regarding reactants and their mixture after isothermal processes at given reaction temperatures may benefit the understanding of the effect of reaction conditions on the DCA process.^53^ Fig. S1a and S1b, in the ESI,† show the TGA (under N_2_) curves of the PFA, IDA, and the mixture of IDA, PFA and GNPs, using the same molar and weight ratios presented in the Experimental part. Three thermal degradation stages are clearly identified: stage (i) corresponds to the degradation of the PFA; stage (ii) is related to the degradation of the IDA and stage (iii), present only in the IDA–PFA–GNP mixture, is related to the degradation of the functional groups resulting from the DCA reaction. The TGA (under N_2_) curves of the same IDA–PFA–GNP mixture after isothermal processes for 5 h at 180 °C, 200 °C, 220 and 250 °C are shown in Fig. S1c and S1d.† The samples are denoted as IDA–PFA–GNPs-180, IDA–PFA–GNPs-200, IDA–PFA–GNPs-220 and IDA–PFA–GNPs-250, respectively. As depicted in Fig. S1d,† only the degradation stages (ii) and (iii) were identified. The stage (ii) (degradation of the IDA) was identified in IDA–PFA–GNPs-180, -200 and -220. There were two peaks in Fig. S1b† that correspond to stage (ii). This is attributed to the 2-stage-degradation of IDA.^49^ With the increase of the temperature from 180 to 220 °C, the IDA degradation occurs at around 230 °C, which leads to the formation of iminodiacetic anhydride along with a partial decarboxylation process. The second IDA degradation peak also showed a decrease with the increase of the temperature of the isothermal process. In the IDA–PFA–GNPs-250 sample, the stage (ii) is completely absent and only the stage (iii) was identified. These results indicate that despite the high melting point of the IDA, the DCA reaction occurs within the temperature range selected. The increase of the reaction temperature seems to decrease the degradation stage (ii), related to the IDA, which may indicate that the IDA is either degraded and/or converted into the functional groups resulted from the DCA reaction.

**Fig. 7:**
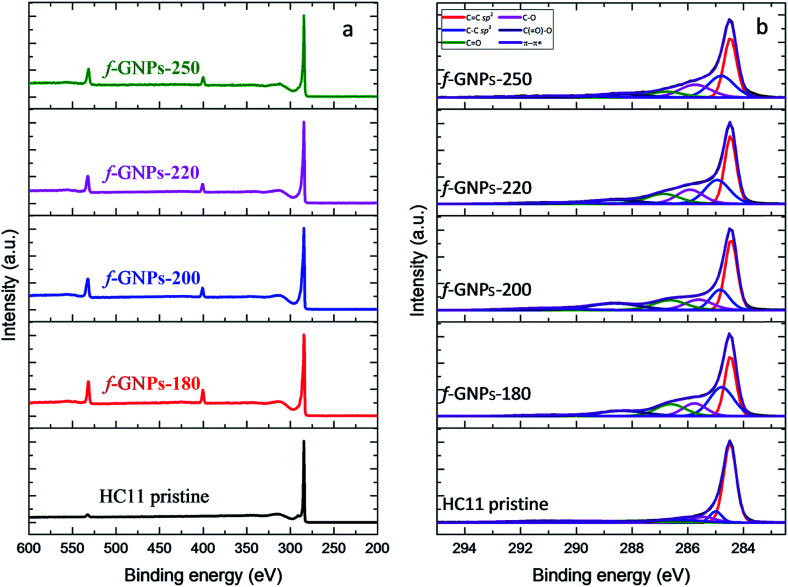
XPS analysis of pristine HC11 and f-GNPs: (a) wide scan spectra; (b) C 1s spectra.

XPS provides a general idea of the elemental composition and bonding states, though any inhomogeneity in the GNPs functionalized *via* this solvent-free method could introduce errors and uncertainties. However, our findings showed that the functionalization of the GNPs is more likely to be homogeneous, according to the titration results. The XPS wide-scan spectra and C 1s spectra of pristine HC11 and f-GNPs synthesized at different temperatures are presented in Fig. 7. In the wide-scan spectra (Fig. 7(a)), a major peak is found at 284 eV, which is attributed to the C 1s energy level. In addition, since the reaction that the GNPs underwent introduced nitrogen-containing functional groups into pristine HC11, a difference in the elemental composition can be observed: in the spectra of f-GNPs, two minor peaks (N 1s, 399 eV; O 1s, 531 eV) are identified, while there is only one (O 1s, 531 eV) in that of pristine HC11.

The C 1s core-level spectra are shown in Fig. 7(b). The contributions of each spectrum are attributed to sp^2^ bonded carbon (284.5 eV, C=C sp^2^), sp^3^ bonded carbon (285.2 eV, C–C sp^3^), alkoxy (286.1 eV, C–O), carbonyl (287.2 eV, C=O), carboxyl (288.9 eV, C(=O)–O) and π–π* shake-up transition (291.1 eV, π–π*), which are presented in red, blue, pink, green, navy and violet colors, respectively. The high resolution C 1s spectrum of pristine HC11 is characterized by the major contribution of C=C sp^2^ and minor contributions of C–C sp^3^, C–O, and C=O and a peak arose from π–π* with low intensity. The potential contribution of C(=O)–O is not observed, while for f-GNPs, contributions of C(=O)–O can be clearly identified, indicating the introduction of carboxyl moieties by the chemical reactions.

The calculation and comparison between the elemental composition (Table 4) and the integration of the area of each contribution (Table 5) provide further information regarding the moiety make-up/content of each GNP.

**Table 4:** Atomic concentration of [C], [N] and [O] in pristine HC11 and the f-GNPs

	At (%)			Ratio		
	[C]	[N]	[O]	[N] : [O]	[N] : [C]	[O] : [C]
HC11	98.14	—	1.86	—	—	0.019
f-GNPs-180	86.52	6.08	7.40	0.82	0.070	0.086
f-GNPs-200	87.85	4.55	7.60	0.60	0.052	0.087
f-GNPs-220	88.64	4.35	7.01	0.62	0.049	0.079
f-GNPs-250	87.89	4.92	7.19	0.68	0.056	0.082

**Table 5:** Integration of normalized contributions of pristine HC11 and the f-GNPs

	HC11	f-GNPs-180	f-GNPs-200	f-GNPs-220	f-GNPs-250
C=C sp^2^	7.55	0.97	2.23	1.35	1.35
C–C sp^3^	1.00	1.00	1.00	1.00	1.00
C(=O)–O	—	0.37	0.88	0.34	0.27
C=O	1.59	0.52	0.86	0.56	0.34
C–O	1.02	0.43	0.76	0.64	0.72
π–π*	1.01	0.11	0.32	0.14	0.28

The integration of contributions of pristine HC11 and f-GNPs was brought into alignment by adjusting the value of C–C sp^3^ to 1.00 and is summarized in Table 5. Ideally, according to Scheme 1, the designed DCA functionalization of HC11 will result in the grafting of a C_4_H_7_NO_2_ moiety to the surface of HC11 flakes, which suggests a [N] : [O] ratio of 1 : 2. Before functionalization, the majority of carbons on the surface of pristine HC11 are sp^2^ bonded ones. The ratio between sp^2^ and sp^3^ carbons can be estimated to be 7.55 : 1.00. Theoretically, this ratio should decrease when functional groups, of which 3 carbons out of 4 are sp^3^ bonded, are introduced by functionalization. Given that IDA undergoes thermal degradation when heated above 210 °C as discussed previously, as well as the consideration of probable side reactions taking place during the functionalization, the actual ratio between [N]/[C] and [O]/[C] could vary. The nitrogen atoms per hundred carbon atoms (NHC) value is 7.0, 5.2, 4.9 and 5.6 for f-GNPs-180, -200, -220 and -250, respectively. The NHC value of f-GNPs-250 is larger than that of f-GNPs-220 and -200, suggesting the larger content of pyrrolidine groups. This is roughly in agreement with the TGA experiment: the content of pyrrolidine groups. The weight loss stage (III) is attributed to the degradation of pyrrolidine groups, thus suggesting that the sequence of pyrrolidine contents of f-GNPs is f-GNPs-250 > -200 > -220 > -180. The NHC values of f-GNPs-200 and -180 suggest that [N] exists in functional groups other than pyrrolidine. According to Table 4, f-GNPs-180 ([O]/[C] = 0.086) and f-GNPs-200 ([O]/[C] = 0.087) should have a similar [O] content. Given that the significant drop in the sp^2^ : sp^3^ ratio of f-GNPs-180 compared to that of f-GNPs-200 suggests a larger quantity of sp^3^ [C]-containing functional groups, *i.e.* a lower degree of unsaturation, there will be more oxygen–carbon single bonds in f-GNPs-180 than in f-GNPs-200. This comparison is supported by Table 5, the [O] containing functional groups of f-GNPs-180 are more in C–O and less in C=O, compared to that of f-GNPs-200, and is generally in agreement with the titration results (Fig. 5). Furthermore, f-GNPs-180 present a great mismatch between the [N] and [O] content ([N]/[O] = 0.82). This may be attributed to side reactions taking place below 200 °C and generating functional groups containing [N], [C] and probably [O], which can withstand heating up to around 200 °C. The existence of these by-products could also be an explanation to the lower thermal degradation onset of f-GNPs-180 than f-GNPs-200 (Fig. 6(a) and (c)), while the weight loss of the two after decarboxylation of *N*-carboxymethyl-pyrrolidine at 300 °C is almost identical.

The high resolution N 1s spectra of the functionalized GNPs (Fig. S2 in the ESI†) are characterized by a contribution at around 400.4 eV which is related to the C–N bonds. The high resolution O 1s spectra of the f-GNPs (Fig. S2 in the ESI†) showed four distinct contributions: C(=O)–O at 531 eV, C=O at 532.1 eV, C–O at 533 eV and C–OH at 533.8 eV. As depicted in Table S2 in the ESI,† the maximum atomic concentration of carboxyl groups (C(=O)–O) is identified in the f-GNPs-200. Furthermore, the atomic concentration of phenolic groups (C–OH) decreases from f-GNPs-180 to f-GNPs-220 and increases for the f-GNPs-250, in agreement with the titration results.

## Conclusions

In summary, the functionalization of HC11 *via* solid-state DCA was performed at selected temperatures of 180, 200, 220 and 250 °C. XRD, Raman spectroscopy, SEM and the electrical conductivity characterization of the pristine HC11 material and the resulting f-GNPs suggested that DCA reactions did not significantly affect the morphological and structural properties of the GNPs. Based on quantitative reactions between acids and bases, Boehm titration experiments can be performed using a simple experimental set-up, sample preparation, operational procedure and data analysis. Such simplicity should drive the application of this method to fast and reliable functionality determination of oxygen-containing groups in functionalized graphene products. Studies regarding functional groups on the surface of f-GNPs were performed by adopting back titration, TGA and XPS. f-GNPs synthesized at 200 °C gave the highest yield of –COOH functional groups. The DCA reaction demonstrated here is found to be highly temperature sensitive. By carefully controlling the reaction temperature around 200 °C, side reactions as well as the undesirable thermal degradation of IDA and the decarboxylation of successfully grafted –COOH groups can be suppressed.

## Conflicts of interest

There are no conflicts to declare.

## Supplementary Material

NA-001-C8NA00280K-s001
