# Supplementary material for: Surface functionality analysis by Boehm titration of graphene nanoplatelets functionalized via a solvent-free cycloaddition reaction
Source: Nanoscale Adv. 2019 Jan 18;1(4):1432–41. doi: 10.1039/c8na00280k (PMC9417147; doi:10.1039/c8na00280k)
Supplement: NA-001-C8NA00280K-s001 [file NA-001-C8NA00280K-s001.pdf]

Electronic Supplementary Material (ESI) for Nanoscale Advances.
This journal is © The Royal Society of Chemistry 2019

Electronic supplementary information

Surface functionality analysis by Boehm titration of graphene nanoplatelets functionalized via a solvent-free cycloaddition reaction

He Ren,[a]† Eunice Cunha,[b]† Quanji Sun,[a] Zheling Li,[b] Ian A. Kinloch,[b] Robert J. Young,[b*] and Zhaodong Fan[a*]

[a] Beijing Institute of Aeronautical Materials (BIAM), Beijing, China

[b] National Graphene Institute and School of Materials, University of Manchester, Manchester M13 9PL, UK

*Corresponding author: robert.young@manchester.ac.uk

fanzhaodong99@sohu.com

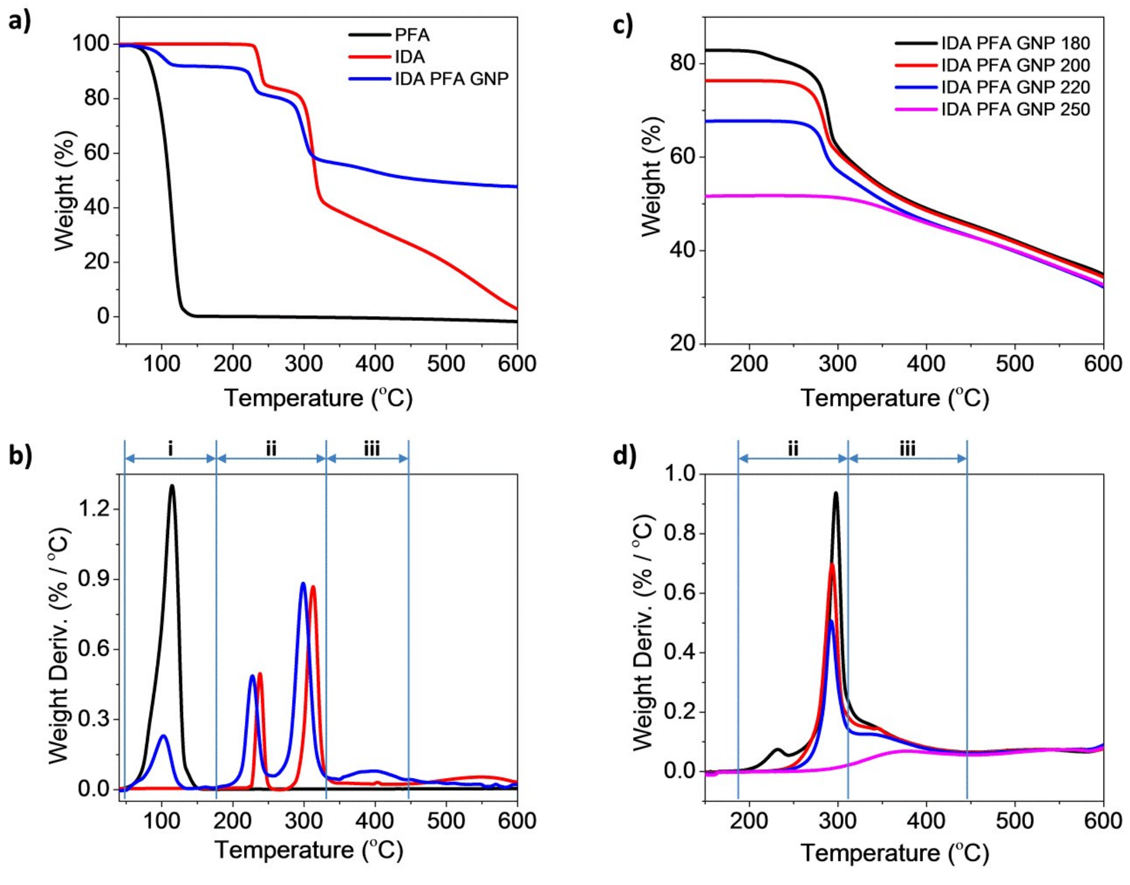

Figure S 1: Thermal gravimetric analysis under an N_2 stream of: a) PFA, IDA and IDA PFA GNP mixture; b) IDA PFA GNP mixture after isothermal processes of 5h at 180 °C, 200 °C, 220 °C and 250 °C; c) weight derivative against temperature of a); d) weight derivative against temperature of b).

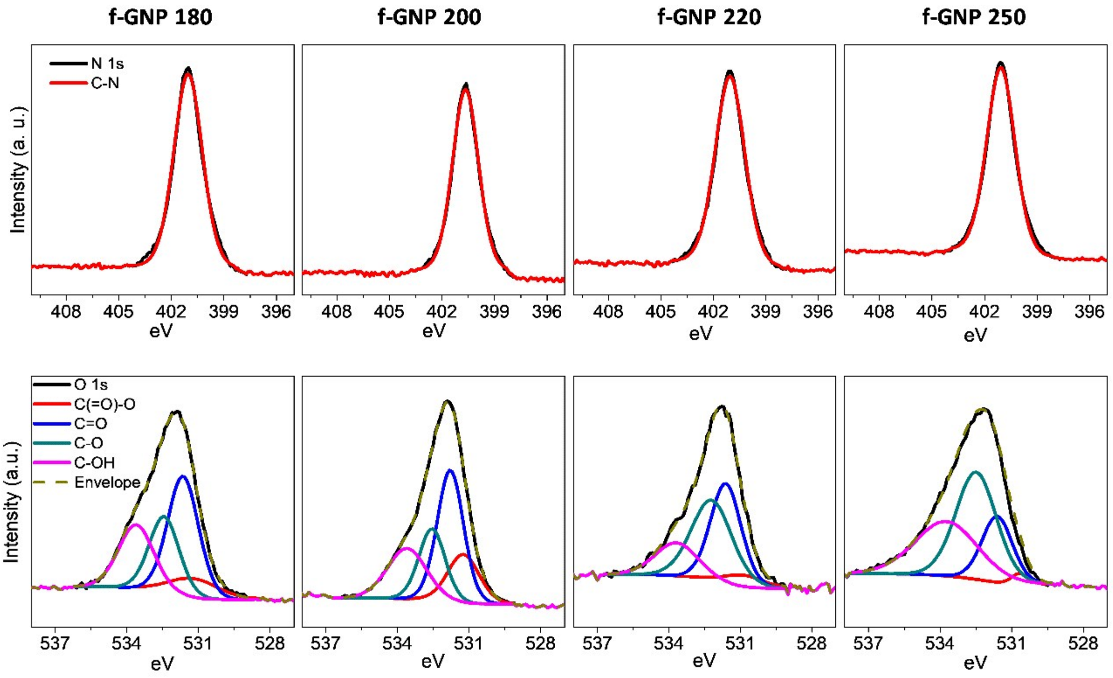

Figure S 2: N 1s and O1s high resolution spectra of the f-GNPs

Table S 1: Atomic concentration (%) of the O 1s components for the f-GNPs

	**C(=O)-O**	**C=O**	**C-O**	**C-OH**
**f-GNP 180**	10.29	40.47	24.77	24.47
**f-GNP 200**	17.35	39.19	2.19	22.27
**f-GNP 220**	5.59	37.37	39.81	17.22
**f-GNP 250**	1.71	20.20	43.75	34.34
